# Exploring the Antiparasitic Activity of the Sea Cucumber *Isostichopus* sp. aff. *badionotus* From the Northern Coast of Colombia Against *Trypanosoma cruzi*

**DOI:** 10.1155/tswj/2849694

**Published:** 2026-06-29

**Authors:** Manuel E. Taborda-Martínez, Ricardo Altamar-Ibáñez, Yeray A. Rodríguez-Núñez, Ericsson Coy-Barrera, Adriana Rodríguez-Forero, Jorge Soto-Delgado, Lisandro Pacheco-Lugo, Mitchell Bacho, Fabián Espitia-Almeida

**Affiliations:** ^1^ Facultad de Ciencias Básicas y Facultad de Ingeniería, Universidad del Magdalena, Santa Marta, Colombia, unimagdalena.edu.co; ^2^ Facultad de Ciencias Básicas, Programa de Biología, Universidad del Atlántico, Puerto Colombia, Colombia, uniatlantico.edu.co; ^3^ Laboratorio de Síntesis y Reactividad de Compuestos Orgánicos, Departamento de Ciencias Químicas, Facultad de Ciencias Exactas, Universidad Andrés Bello, Santiago, Chile, unab.cl; ^4^ Bioorganic Chemistry Laboratory, Universidad Militar Nueva Granada, Cajicá, Colombia, umng.edu.co; ^5^ Departamento de Ciencias Químicas, Facultad de Ciencias Exactas, Universidad Andrés Bello, Viña del Mar, Chile, unab.cl; ^6^ Centro de Investigaciones en Ciencias de la Vida, Facultad de Ciencias Básicas y Biomédicas, Universidad Simón Bolívar, Barranquilla, Colombia, unisimon.edu.co

**Keywords:** docking studies, sea cucumber, sulfated sterols, trypanocidal activity, UHPLC–MS

## Abstract

Chagas disease, caused by *Trypanosoma cruzi*, remains a neglected tropical infection with limited therapeutic options. Sea cucumbers are recognized as rich sources of bioactive sulfated metabolites with potential antiparasitic properties; however, information on the chemistry and trypanocidal activity of Caribbean *Isostichopus* species is still scarce. In this study, the trypanocidal activity of solvent fractions from *Isostichopus* sp. aff. *badionotus* was evaluated, together with the chemical characterization and molecular docking analysis of their major metabolites. The methanolic extract, obtained by maceration of lyophilized tissue, was partitioned with *n*‐hexane, dichloromethane, and *n*‐butanol, and the resulting fractions were analyzed by UHPLC–HRMS. The in vitro trypanocidal activity was assessed against *T. cruzi* epimastigotes, and molecular docking studies explored the interactions of five sulfated sterols (**1–5**) with four key enzymes involved in the parasite′s redox metabolism. Chemical profiling revealed that sulfated sterols, mainly cholestane‐ and stigmastane‐type derivatives, predominated in the dichloromethane fraction, which exhibited the strongest trypanocidal activity. Docking results indicated that stigmastane‐type sterols, particularly Compound **5**, showed the most favorable binding energies with all enzyme targets. This study provides the first evidence of trypanocidal activity in *Isostichopus* sp. aff. *badionotus*, highlighting sulfated sterols (**1–5**) as plausible lead compounds for exploring agents against *T. cruzi*.

## 1. Introduction

Chagas disease, caused by the protozoan parasite *Trypanosoma cruzi*, is a significant public health problem in Latin America and remains a leading cause of disability and mortality in affected regions [1]. An estimated 7 million people across 21 Latin American countries are infected, with the Americas reporting ~30,000 new cases and ~12,000 deaths annually, alongside ~9000 congenital infections each year [2, 3]. This pathology is regarded as the most significant neglected disease in Latin America, with epidemiological risk factors closely linked to socioeconomic and cultural conditions [4–6]. In Colombia, approximately 1.2 million people are infected, and 8 million remain at risk [7, 8]. The persistence of the disease is linked to its zoonotic nature, diverse transmission routes, and social determinants such as poverty and rural exposure. Clinically, the disease progresses from an acute phase, often asymptomatic, to a chronic phase that can cause severe cardiac and gastrointestinal complications [5, 9, 10]. Current treatments, benznidazole and nifurtimox, are limited by variable efficacy in chronic infections and frequent adverse effects, underscoring the urgent need for novel, more tolerable therapeutic options [11–16].

Targeting the parasite′s redox metabolism has emerged as a promising strategy in *T. cruzi* drug discovery. Enzymes such as trypanothione reductase (*Tc*TR), trypanothione synthetase (*Tc*TryS), and cysteine synthetase (*Tc*CS) are essential for oxidative stress defense and parasite survival. Inhibitors of these enzymes disrupt the parasite′s redox balance, leading to oxidative damage and apoptosis [17–19]. Likewise, trypanothione reductase has been identified as a key target, as its inhibition could impair *T. cruzi*’s ability to counteract cellular damage caused by reactive oxygen species [20, 21]. Molecular docking and structure‐based design approaches have shown that small molecules, including sterol derivatives, can effectively bind these enzymes, supporting their development as chemotherapeutic leads.

Natural products have shown great promise in the search for new anti‐Chagas compounds, owing to their structural diversity and bioactivity [22–24]. In this context, marine organisms have emerged as a pivotal focus in natural product chemistry, as they produce a unique and diverse array of bioactive compounds [25]. Alongside their increasing global consumption for nutritional and health benefits, there is a growing interest in analyzing and characterizing their chemical composition.

Among the diverse marine taxa, holothurians—commonly known as sea cucumbers—represent a noteworthy group. These echinoderms, comprising roughly 2000 species, are distributed worldwide, with some cultivated in aquaculture and consumed as food [26]. Sea cucumbers are benthic, free‐living invertebrates that feed on organic matter present in sediments or suspended in the water column. They are known to produce an array of secondary metabolites, notably saponins, glycosylated or sulfated sterols (STs), triterpenes, and long‐chain lipids—some unique to their own metabolism and others derived from dietary sources. Over recent decades, extracts from various sea cucumber species have demonstrated multiple biological activities, including antimicrobial, anticancer, hemolytic, cytostatic, and immunomodulatory effects. Likewise, several pharmacological activities have been attributed to specific isolated compounds [27–29].

The genus *Isostichopus* comprises sea cucumber species of high ecological and economic importance in the tropical Western Atlantic. Recent taxonomic studies have identified *Isostichopus* sp. aff. *badionotus* as a distinct entity based on habitat distribution, morphological characteristics, reproductive biology, and genetic differentiation [30]. Despite the recognized ecological value and the increasing pharmacological interest in sea cucumbers as sources of bioactive marine metabolites, the chemical composition and biological properties of this taxon remain virtually unexplored. A recent study reported the first insights into the chemical profiling and antiparasitic potential of *Isostichopus* sp. aff. *badionotus*, revealing the presence of lipid‐derived compounds as major constituents [31]. Building upon this precedent, the present study is aimed at: (i) evaluating the in vitro trypanocidal activity of solvent fractions of different polarity obtained from *Isostichopus* sp. aff. *badionotus* collected along the northern Caribbean coast of Colombia; (ii) chemically characterizing the active fractions using UHPLC–MS/MS; and (iii) exploring, through in silico molecular docking, the interactions and potential molecular targets of the main constituents against key enzymes involved in the redox metabolism of *T. cruzi*.

## 2. Materials and Methods

### 2.1. Chemical and General Experimental Procedures

All reagents and solvents were of analytical grade. Methanol (MeOH), *n*‐butanol (BuOH), dichloromethane (DCM), and *n*‐hexane (Hex) were purchased from Merck Millipore (Merck, Hohenbrunn, Germany).

### 2.2. Collection of Animal Material

**Figure 1:**
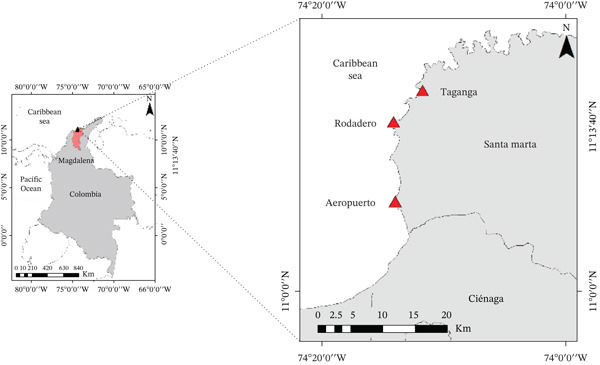
Sampling location of studied sea cucumber specimens in the Caribbean Sea, northern Colombia.

Specimens of *Isostichopus* sp. aff. *badionotus* were manually collected during an annual cycle at three sites along the northern Caribbean coast of Colombia: 1) Rodadero (11°13 ^′^22.73 ^″^ N, 74°13 ^′^32.59 ^″^ W), 2) Airport (11°07 ^′^10 ^″^ N, 74°13 ^′^50 ^″^ W), and 3) Taganga (11°16 ^′^04.6 ^″^ N, 74°11 ^′^46.9 ^″^ W), at depths ranging 5–10 m (Figure 1). Only adult individuals, confirmed by ichthyometer measurements and weight determinations, were selected. Specimens were placed in sealed polyethylene bags, stored on ice (4°C), and transported to the laboratory. Taxonomic identification followed Vergara et al. [32]. In addition, a specimen was set up for inclusion in the collection of Biological Sciences of the University of Magdalena in Santa Marta, Colombia (CBUMAG:ECH:00001, CBUMAG:ECH:00002, CBUMAG:ECH:00003).

### 2.3. Extraction Procedure

Whole animals were cut into small pieces, frozen at −80°C, and lyophilized. Dried tissue (0.6 kg) was exhaustively macerated in MeOH (6 L) for 3 days at room temperature, filtered, and concentrated under reduced pressure. The crude MeOH extract was then partitioned successively with Hex, DCM, and BuOH to yield fractions (3.6, 6.6, 8.6 g, respectively) of increasing polarity.

### 2.4. Parasite Culture

The antitrypanosomal activity of sea cucumber fractions was evaluated against *T. cruzi* (strain Dm28c) in the epimastigote stage. This developmental form was selected as a primary screening model due to its suitability for rapid, reproducible, and cost‐effective bioassays, as well as previously reported correlations between in vitro inhibitory activity against epimastigotes and in vivo efficacy against bloodstream trypomastigotes [33–35]. In addition, epimastigotes can be cultured axenically without the need for host cell systems or high‐level biosafety conditions, making them particularly appropriate for preliminary evaluation of crude extracts. Parasites were maintained in liver infusion tryptose (LIT) medium supplemented with 10% heat‐inactivated fetal bovine serum (FBS), 2% hemin, and 100 *μ*g mL^−1^ penicillin, and incubated at 28°C until reaching a density of 2.5 × 10^5^ cells mL^−1^, as determined using a Neubauer chamber.

### 2.5. In Vitro Inhibitory Assay for Antiepimastigotes of *T. cruzi*

The inhibitory activity of each fraction was determined using the MTT [3‐(4,5‐dimethylthiazol‐2‐yl)‐2,5‐diphenyltetrazolium bromide] colorimetric assay in 96‐well microplates [36, 37]. Stock solutions (100 mg mL^−1^) were prepared in dimethyl sulfoxide (DMSO) and diluted with phosphate‐buffered saline (PBS) to working solutions of 2 mg mL^−1^. Each well received 100 *μ*L of parasite inoculum and 100 *μ*L of fraction solution, yielding final concentrations of 0.001–1 mg mL^-1^. Plates were incubated at 28°C for 24, 48, 72, 96, and 120 h. At each time point, 50 *μ*L of MTT solution (5 mg mL^−1^) was added, followed by 4 h incubation. The medium was removed, and formazan crystals were solubilized in 100 *μ*L DMSO for 15 min before measuring absorbance at 570 nm (CLARIOstar Plus, BMG LABTECH). Nifurtimox (10 *μ*g mL^−1^) served as a positive control, and DMSO (≤ 1%) as the negative control. All assays were performed in triplicate and repeated independently, with a coefficient of variation < 20% (each replicate included four treatments, and a total of 12 independent experiments were conducted for each concentration).

### 2.6. Statistical Analysis

Data are expressed as mean ± SD from at least three independent experiments. Statistical significance was determined by one‐way ANOVA followed by Tukey′s post hoc test (GraphPad Prism 9.3), with *p* < 0.05 considered significant.

### 2.7. Chemical Composition Analysis

The resulting dried MeOH extract, and each dried fraction (Hex, DCM, and BuOH) were dissolved in HPLC‐grade MeOH and transferred to HPLC vials. Chemical profiling was performed on a Compact QTOF–MS coupled to an Elute UHPLC system (Bruker Daltonik GmbH, Bremen, Germany) with a Kinetex C18 column (Phenomenex) (2.1 × 100 mm, 1.7 um) maintained at 40°C. Mobile phases consisted of (A) water +0.1% formic acid and (B) acetonitrile +0.1% formic acid, with the following gradient: 0 min, 88% A; 12.5 min, 70% A; 14.5 min, 40% A; 19 min, return to initial conditions. The flow rate was 0.5 mL min^−1^, the injection volume was 5 *μ*L, and the total run time was 19 min. Mass spectra were acquired in both positive and negative electrospray ionization (ESI) modes over *m*/*z* 120–1800, involving the following parameters: capillary temperature of 200°C, a capillary voltage of 2.0 kV, a dry gas flow rate of 8 L/min, and a nebulizer pressure of 2 bar. Data were processed in MetaboScape 4.0 (Bruker). Compound annotation was performed by combining high‐resolution monoisotopic mass measurements with MS/MS fragmentation patterns and chromatographic retention times. The resulting data were compared against established spectral libraries, including MS‐DIAL, the MassBank of North America (MoNA), and LipidBlast, to support accurate metabolite identification. Confidence levels for HRMS‐based metabolite identification were assigned following previously established reporting standards (Levels 1–4) [38].

### 2.8. Molecular Docking

Optimal binding orientations and conformations of the compounds within the target proteins were determined through molecular docking calculations using AutoDock 4 [39]. The atomic coordinates of the proteins were obtained from the X‐ray crystal structures of cysteine synthetase (*Tc*CS; PDB ID: 8B9Y) [40]. trypanothione reductase (*Tc*TR; PDB ID: 1BZL) [41], and spermidine synthase (*Tc*SpdSyn; PDB ID: 5Y4P) [42], as deposited in the RCSB Protein Data Bank. A homology model of trypanothione synthetase (*Tc*TryS) was generated from the UniProt sequence Q9GT49 using PyMOD 3.0 (Department of Biochemical Sciences, Sapienza University of Rome, Rome, Italy) [43] and MODELLER 10.5 [44] and validated using PROCHECK [45]. Protein–ligand interactions were analyzed with the protein–ligand interaction profiler (PLIP) [46] and visualized in PyMOL molecular graphics system.

## 3. Results

### 3.1. Antiparasitic Activity

**Figure 2:**
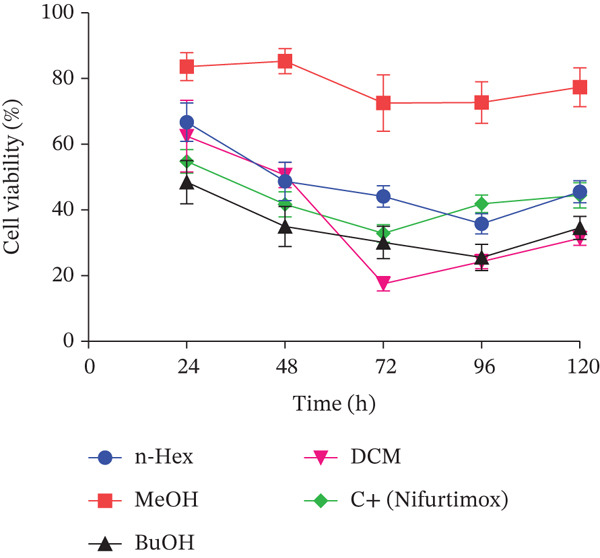
Viability of *Trypanosoma cruzi* epimastigotes (strain Dm28c) after exposure to fractions (*n*‐butanol [BuOH], dichloromethane [DCM], *n*‐hexane [Hex], and methanol [MeOH]) obtained from *Isostichopus* sp. aff. *badionotus* at a test concentration of 1.0 *μ*g/*μ*L between 24 and 120 h.

The present study investigated the antiparasitic activity of the fractions from the sea cucumber *Isostichopus* sp. aff. *badionotus* against *T. cruzi* epimastigotes (strain Dm28c) at a screening concentration of 1.0 *μ*g/*μ*L. The DCM, BuOH, and Hex fractions were the most effective in significantly reducing parasite development between 24 and 120 h (Figure 2), with the most effective response at 72 h.

**Figure 3:**
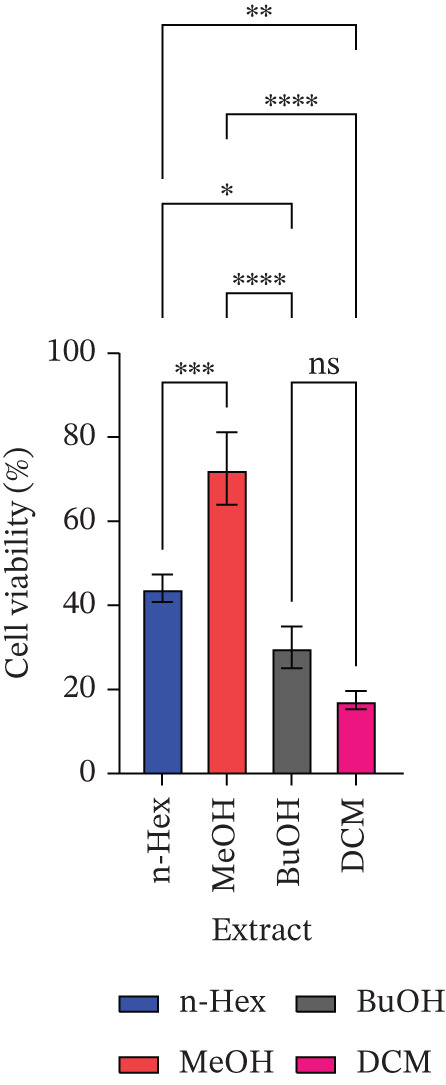
Antiparasitic effect of the fractions (*n*‐butanol [BuOH], dichloromethane [DCM], *n*‐hexane [Hex], and methanol [MeOH]) obtained from *Isostichopus* sp. aff. *badionotus* against the epimastigotes of *T. cruzi* (strain Dm28c) at a test concentration of 1.0 *μ*g/*μ*L for 72 h of exposure. ns: not significant. ∗(*p* < 0.03), ∗∗(*p* < 0.002), ∗∗∗(*p* < 0.001), ∗∗∗∗(*p* < 0.0001).

The highest antiparasitic potential against *T. cruzi* epimastigotes (strain Dm28c) was obtained after 72 h of treatment. During that period, the significance of pharmacological potency between the fractions was evaluated by one‐way ANOVA, followed by a post hoc Tukey test (Figure 3), which found that the DCM and butanol fractions have similar potency against *T. cruzi* epimastigotes (strain Dm28c) (*p* > 0.05, ns). The statistical analysis also showed that the DCM and butanol fractions were significantly more potent than the rest of the fractions, with cell viabilities of 17.5*%* ± 2.16*%* and 30.1*%* ± 4.9*%* (*p* < 0.0001), respectively. Additionally, the MeOH extract was the least potent, presenting a cell viability of 72.5*%* ± 8.6*%*.

The IC_50_ (half‐maximal inhibitory concentration) was calculated at 72 h by using the probit method for the tested fractions (i.e., DCM, BuOH, and Hex), since they exhibited a response higher than 50% at the screening concentration. Thus, the IC_50_ values and the probit model parameters, such as 95% confidence intervals, slope, and *R* ^2^ are presented in Table 1.

**Table 1:** IC_50_ values of *Isostichopus* sp. aff. *badionotus*‐derived tested fractions against *T. cruzi* and probit model parameters.

Fraction^a^	IC_50_ (*μ*g/mL), IC, 95%	Slope, IC, 95%	*R* ^2^
DCM	565.1 (553.2–578.0)	8.0 (6.7–9.6)	0.91
BuOH	691.6 (658.3–728.9)	2.3 (2.0–2.7)	0.93
Hex	737.6 (712.8–766.0)	4.8 (3.9–5.9)	0.90
MeOH	> 1000	—	—

*Note:* IC, 95: 95% confidence interval, *R* ^2^: coefficient.

^a^ *n*‐Butanol (BuOH), dichloromethane (DCM), *n*‐hexane (Hex), and methanol (MeOH).

### 3.2. Chemical Characterization of *Isostichopus* sp. aff. *badionotus* Fractions

The UPLC–HRMS‐based metabolite profiling of the fractions derived from *Isostichopus* sp. aff. b*adionotus* revealed chemical patterns consistent with those previously described [31], involving a broad diversity of lipid‐derived metabolites. The annotation of secondary metabolites in both positive and negative ion modes was performed by comparing accurate high‐resolution monoisotopic masses, MS/MS fragmentation spectra, and chromatographic retention times against the MS‐DIAL, MoNA, and LipidBlast databases, ensuring reliable metabolite identification. The overall chemical profile confirmed the predominance of lipid classes such as lysophospholipids, phosphatidylcholines (PC), and sulfated STs [31]. Among the identified metabolites, the STs represented the most abundant and chemically distinctive constituents within the active fractions. In this regard, STs accounted for 2.16% of the total metabolites in the Hex fraction, 17.46% in the DCM fraction, and 36.52% in the BuOH fraction, while they were not detected in the methanolic extract. These results underscore the enrichment of sulfated STs in the medium‐ to high‐polarity fractions of *Isostichopus* sp. aff. *badionotus*. Thus, Table 2 summarizes the percentage distribution of the identified STs in the negative ion mode across the test fractions, confirming their predominance in the DCM and BuOH fractions. This metabolite family is represented by five sulfated STs, which constitute the major secondary metabolites of *Isostichopus* sp. aff*. badionotus*. Among these compounds, STs **1**, **2**, and **3** correspond to cholestane‐type STs, whereas **4** and **5** are stigmastane‐type derivatives.

**Table 2:** Sulfated sterols detected in the *Isostichopus* sp. aff. *badionotus*‐derived fractions.

Compound	Structure	Name	MF^a^	AM^b^	RT^c^	Hex^d^	DCM^d^	BuOH^d^	MeOH^d^
**1**		Cholesterol sulfate	C_27_H_46_O_4_S	466.3117	10.89	0.48	4.54	9.23	0.00
**2**		24‐Methylene cholesterol sulfate	C_28_H_46_O_4_S	478.3117	11.75	0.45	0.15	8.72	0.00
**3**		24‐Methyl cholesterol sulfate	C_28_H_48_O_4_S	480.3273	12.15	0.43	1.13	6.89	0.00
**4**		Stigmasterol sulfate	C_29_H_48_O_4_S	492.3273	11.31	0.30	5.14	5.78	0.00
**5**		Stigmast‐7‐enol sulfate	C_29_H_48_O_4_S	492.3273	12.64	0.50	6.50	5.90	0.00

^a^MF, molecular formula; AM, accurate mass [M].

^b^Retention time (min).

^c^RT, retention time.

^d^Relative percentage (%) of each compound in the test fractions (*n*‐butanol [BuOH], dichloromethane [DCM], *n*‐hexane [Hex], and Methanol [MeOH]).

In the Hex fraction, STs are present in similar percentages, with stigmast‐7‐enol sulfate (**5**, 23.15%) and cholesterol sulfate (**1**, 22.22%) being the major compounds. The content in the DCM fraction is dominated by STs derived from the stigmastane skeleton (**5**, 37.23% and **4**, 29.44%) and, to a lesser extent, cholestane derivatives (**1**, 26.00%; **3**, 6.47%; and **2**, 0.86%). In contrast, the butanolic fraction presents a higher percentage of sulfated STs associated with the cholestane skeleton (**1**, 25.27%; **2**, 23.88%; and **3** 18.87%) than their analogues from the stigmastane nucleus (**5**, 16.16% and **4**, 15.83%). Considering the global percentages and linking with the individual percentages of each sulfated sterol, the composition of these in the fractions can be categorized, in terms of the abundance of STs, as DCM > BuOH > Hex.

### 3.3. Molecular Docking Studies

**Figure 4:**
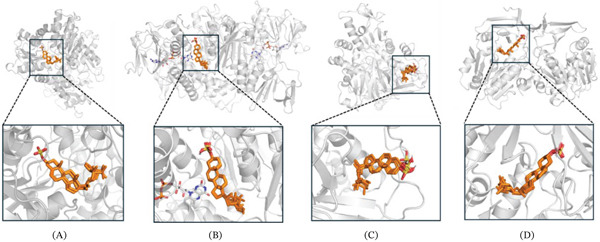
The most favored poses of sterol compounds within the active site of test enzymes, defined by molecular docking. (A) Cysteine synthetase, (B) trypanothione reductase, (C) trypanothione synthetase, and (D) spermidine synthase.

To investigate the probable binding modes of the sulfated sterol derivatives, molecular docking analyses were performed to identify potential molecular targets. In this context, the interactions of the STs were examined with four key enzymes representative of the *T. cruzi* trypanothione‐dependent redox metabolism (Figure 4), given previous evidence that sterol‐like compounds can interfere with this essential biochemical pathway [47, 48].

In this context, four key enzymes involved in the *T. cruzi* redox metabolic pathway were selected as potential therapeutic targets, such as cysteine synthetase (*Tc*CS), trypanothione reductase (*Tc*TR), trypanothione synthetase (*Tc*TryS), and spermidine synthase (*Tc*SpdSyn). Binding poses and docking scores were evaluated using AutoDock 4‐based scoring functions. As summarized in Table 3, all five STs exhibited favorable binding energies toward the four enzymes. Notably, STs **4** and **5** showed the most favorable docking scores, consistent with their higher relative abundance in the DCM fraction. These findings suggest that the trypanocidal activity observed for this fraction might be attributed mainly to the stigmastane‐type sulfated STs.

**Table 3:** Binding affinity of sulfated sterols according to the Autodock score function.

Compound	Name	Docking Scores (kcal/mol)			
		*Tc*CS	*Tc*TR	*Tc*TryS	*Tc*SpdSyn
**1**	Cholesterol sulfate	−9.74	−8.61	−10.05	−10.40
**2**	24‐methylene cholesterol sulfate	−10.02	−9.02	−10.30	−9.95
**3**	24‐methyl cholesterol sulfate	−9.95	−9.03	−10.23	−10.11
**4**	Stigmasterol sulfate	−10.62	−9.28	−10.50	−10.81
**5**	Stigmast‐7‐enol sulfate	−11.29	−9.38	−10.78	−10.91

**Figure 5:**
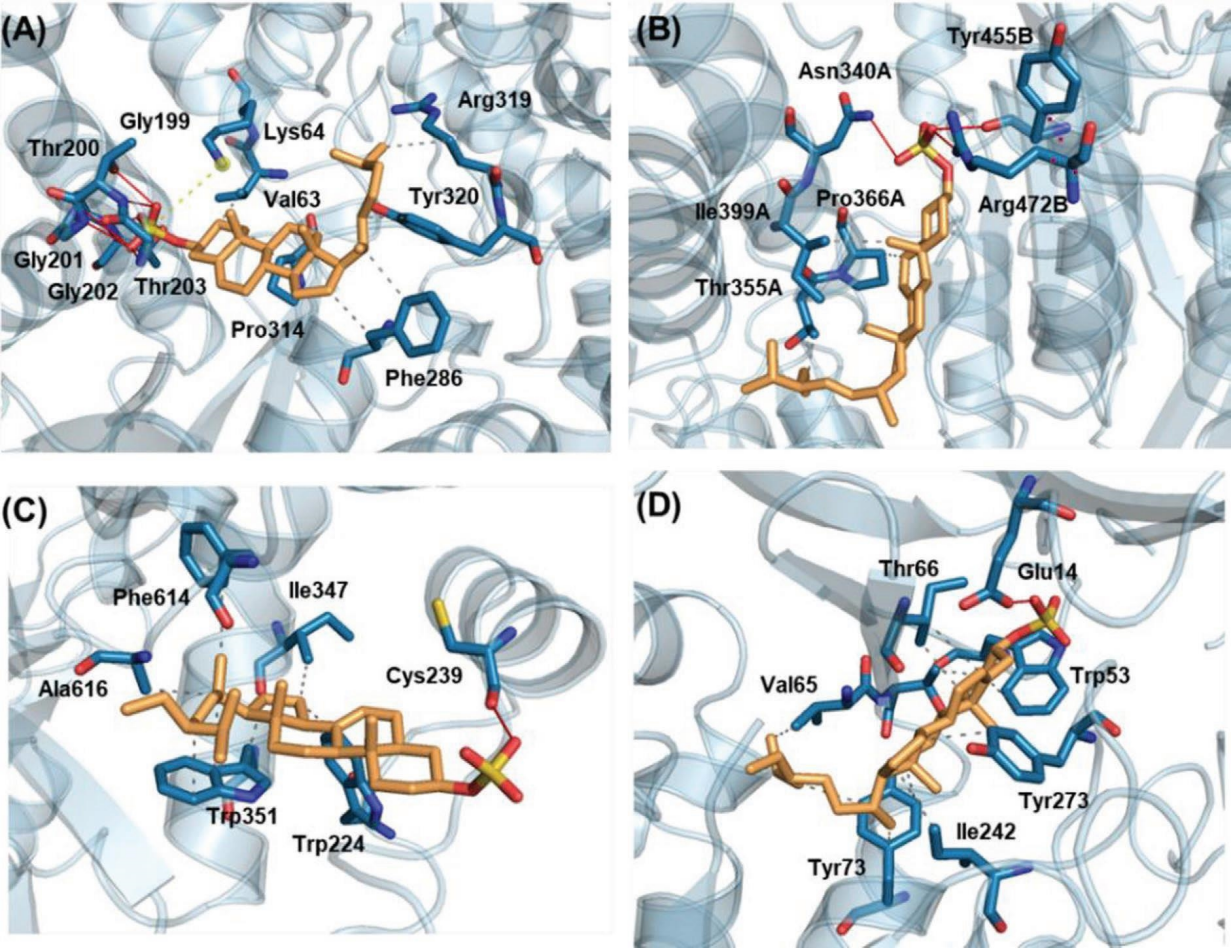
Proposed binding mode for sulfated sterol **5** at the active site of (A) cysteine synthetase, (B) trypanothione reductase, (C) trypanothione synthetase, and (D) spermidine synthase.

Figure 5 shows the interactions between ST **5**, the highest top‐ranked compound, and the different amino acid residues present in the active sites of the test enzymes. This pose is consistent with the values obtained for the percentage composition of the DCM fraction and with the trypanosomicidal activity values obtained for this fraction.

The most abundant compound, ST **5**, exhibited the strongest binding affinity toward cysteine synthetase (*Tc*CS). Molecular docking revealed favorable *H*‐bond interactions between the sulfate moiety and residues Gly199, Thr200, Gly201, Gly202, and Thr203 (2.4 Å) (Figure 5A). In addition, a salt‐bridge interaction between compound **5** and Lys64 (2.4 Å) further supports its affinity toward *Tc*CS. In addition, hydrophobic interactions were also predicted between the sterol′s hydrophobic region and residues Leu63, Leu399, and Phe396, contributing to overall complex stability. Regarding trypanothione reductase (*Tc*TR; PDB ID: 1BZL), the STs were found to interact favorably within an intermediate region between these sites (Figure 5B). The docking model suggests *H*‐bonding between the sulfate group and residues Asn340 (2.4 Å), Tyr455, and Arg472. However, the binding scores for the analyzed compounds were lower than those observed for the other targets.

The binding modes of the STs with trypanothione synthetase (*Tc*TryS) were also evaluated. In agreement, all STs in this study displayed comparable docking scores, with compound **5** showing the most favorable docking score (−10.78 kcal/mol; Table 3). The binding mode was characterized by hydrophobic interactions involving residues Trp224, Ile347, Trp351, Phe614, and Ala616. Additionally, a hydrogen bond between the sulfate group and Cys239 (2.4 Å) contributed to further stabilization of the ligand within the active site (Figure 5C). Similarly, for spermidine synthase (*Tc*SpdSyn; PDB ID: 5Y4P), hydrophobic interactions predominated. Compound **5** exhibited the best affinity (−10.91 kcal/mol; Table 3), stabilized by interactions with residues Trp53, Val65, Thr66, Tyr73, Ile242, and Tyr273 (Figure 5D). A *H*‐bond with Glu14 (2.4 Å) was also identified.

## 4. Discussion

The consumption of sea cucumber species has increased considerably in recent years due to their nutritional value and bioactive potential as functional foods. This growing interest underscores the importance of understanding both their chemical composition and pharmacological properties. In this context, the present study examined the antiparasitic activity of Hex, DCM, and BuOH fractions against *T. cruzi* epimastigotes, obtained from a newly described Caribbean sea cucumber, *Isostichopus* sp. aff. *badionotus*. Chemical profiling revealed that sulfated STs were the predominant metabolites across the active fractions, representing the most abundant and consistent compound class. Furthermore, the biological assays demonstrated that these fractions effectively reduced *T. cruzi* epimastigote viability, supporting their potential pharmacological relevance. Several of the metabolites identified in *Isostichopus* sp. aff. *badionotus*, particularly cholesterol sulfate **1**, cholestane derivatives (**2** and **3**), and stigmastane‐type STs (**4** and **5**), have previously been reported in other sea cucumber species. For instance, cholesterol sulfate was identified as the major metabolite in *Eupentacta scamatrix*, followed by cholestane‐ and stigmastane‐type STs with moderate abundances [49]. Similarly, *Psolus fabricii* exhibits high levels of these STs, except for stigmastane **5**, which occurs in lower concentrations [50]. However, previous studies describing these metabolites in echinoderms have focused mainly on their structural diversity, with limited attention to their pharmacological or antiparasitic potential. To our knowledge, this is the first report linking sulfated STs from *Isostichopus* species with activity against *T. cruzi*. These findings warrant further investigation to validate the functional significance of these compounds and to explore their potential contribution to the health‐promoting properties attributed to sea cucumber consumption.

Sulfated STs, particularly those sulfated at the C3 position, are widely distributed in marine organisms and have been associated with diverse biological activities, including anti‐inflammatory, cytotoxic, and antimicrobial effects [51–53]. In addition to STs, various lipid classes such as PC, phosphatidylethanolamines (PE), lysophosphatidylcholines (LPC), lysophosphatidylethanolamines (LPE), lysophosphatidylinositols (LPI), and sphingomyelins (SM) have been characterized in sea cucumbers [31] notably *Stichopus japonicus*, through mass spectrometry analysis [54]. Likewise, in *Bohadschia marmorata*, *Parastichopus californicus*, *Holothuria mexicana*, *Cucumaria frondosa*, *Holothuria poli*, and *Isostichopus fuscus*, phospholipid species from the PC‐O, PC, PE, and PS classes have been identified with differential abundances [40]. When compared with these previous reports, *Isostichopus* sp. aff. *badionotus* displays a similarly diverse lipid profile, suggesting that this species, belonging to the families Cucumariidae, Holothuriidae, and Stichopodidae, is a promising source of secondary metabolites with broad biological potential.

In the chemical characterization of the newly identified sea cucumber *Isostichopus* sp. aff*. badionotus*, no saponins were detected, which are commonly found in many holothurian species. This absence does not necessarily indicate that saponins are absent; instead, it suggests the need for a more detailed analysis to annotate/identify compounds that exhibit low spectral similarity with the employed databases. Further fractionation of the crude fractions, using either liquid–liquid or liquid–solid partitioning, is also required to simplify the chemical matrix, facilitate chemical/biological screening and compound identification, and enhance the reliability of metabolite annotation.

The antiparasitic activity assays revealed that the DCM and BuOH fractions were significantly more effective in reducing the viability of *T. cruzi* epimastigotes (strain Dm28c) than the other fractions (*p* <0.0001; Figure 3). This effect appears to be associated with the chemical composition and relative abundance of secondary metabolites within these fractions. As demonstrated by the chemical characterization, sulfated STs were the predominant metabolites in both the DCM and BuOH fractions, suggesting that these compounds are primarily responsible for the observed trypanocidal activity. To our knowledge, this represents the first report of sulfated STs as potential inhibitors of *T. cruzi* epimastigote viability. This hypothesis is supported by previous findings describing structurally related triterpenes and sterol derivatives with potent antiparasitic properties. For instance, lupeol acetate has been reported as an active compound against *T. cruzi* [55], and synthetic modifications of pregnenolone, which involve the formation of 1,2,3‐triazole moieties, while preserving the cyclopentanoperhydrophenanthrene core typical of STs, have yielded derivatives with up to sixfold greater inhibitory activity than nifurtimox, the current reference drug [56]. These examples reinforce the potential antiparasitic effects of compounds containing the sterol nucleus, consistent with the activity observed for the sulfated STs identified in our study. Regarding lethality over time, both the fractions and the positive control (nifurtimox) remained active for up to 72 h (Figure 3). Beyond this period, parasite viability increased substantially, suggesting that the activity of the fractions′ metabolites may be unstable or subject to degradation over time [57]. Additionally, the complex biology of *T. cruzi*, characterized by extensive genetic heterogeneity within strains, may contribute to adaptive responses and clonal variation that reduce susceptibility to the stress induced by either the fractions or the control compound [58–60].

The chemical and biological results presented in this study provide new insights into the trypanocidal potential of the sea cucumber *Isostichopus* sp. aff. *badionotus*. The DCM fraction, enriched in sulfated STs, exhibited the strongest inhibitory effect against *T. cruzi* epimastigotes, in agreement with the abundance and diversity of these metabolites. Previous studies have shown that marine STs, particularly sulfated derivatives, possess diverse biological activities [61, 62], possibly mediated by interactions with enzymes involved in cellular redox homeostasis. The molecular docking results obtained here support this plausible mechanism of action. Among the five identified STs, Compound **5**, the most abundant in the active fraction, showed the strongest predicted affinity for *Tc*CS [40]. This enzyme plays a crucial role in thiol‐dependent redox metabolism in *T. cruzi* and has recently been recognized as a promising target for terpenoid‐based inhibitors [55]. The observed *H*‐bonding between the sulfate moiety of Compound **5** and residues Gly199‐Thr203, along with salt‐bridge and hydrophobic interactions, indicated a strong and specific stabilization within the *Tc*CS binding pocket. These findings suggest that *Tc*CS may represent a key target mediating the trypanocidal effect of these marine‐derived STs. In contrast, the interaction of the STs with *Tc*TR [41], was comparatively weaker. Although TcTR is a well‐established target for antitrypanosomal agents [63], the compounds analyzed in this study bound preferentially to an intermediate region between the mepacrine and Z‐sites, rather than to the canonical active site. This alternative binding mode may reflect a noncompetitive inhibition mechanism, consistent with the enzyme′s known regulatory dynamics. Regarding *Tc*TryS, a key enzyme in the biosynthesis of trypanothione, all STs showed similar docking energies, with Compound **5** exhibiting the highest affinity. The interactions were dominated by hydrophobic contacts with six residues, complemented by *H*‐bonding with Cys239. These findings are consistent with previous in silico studies demonstrating the strong binding of terpenoid derivatives to *Tc*TryS [64]. A comparable trend was observed for *Tc*SpdSyn, where Compound **5** showed the best affinity, stabilized mainly by hydrophobic interactions with six residues, as well as an *H*‐bond with Glu14. The combined docking evidence suggests that sulfated STs act through a multitarget mechanism, preferentially interacting with *Tc*CS, *Tc*TryS, and *Tc*SpdSyn. These results provide a molecular rationale for the trypanocidal activity observed in the DCM fraction of *Isostichopus* sp. aff. *badionotus*. The high abundance of stigmastane‐type sulfated STs (Compounds **4** and **5**), coupled with their strong predicted affinities for key enzymes in the trypanothione‐dependent redox pathway, suggests that these metabolites may interfere with essential antioxidant defenses of *T. cruzi*. This multitarget interaction profile aligns with the polypharmacological nature often observed in marine natural products and underscores the potential of sulfated STs as lead compounds for developing new antiparasitic agents. Further studies, including enzyme inhibition assays and structure–activity relationship analyses, are warranted to confirm these interactions and explore their therapeutic potential.

Despite its preliminary nature, this study is technically sound and adheres to accepted analytical and experimental standards in marine natural products research, employing validated HPLC–MS methods to characterize the methanolic extract and solvent fractions from *Isostichopus* sp. aff. *badionotus*. The anti‐*T. cruzi* activity of these fractions and their constituents is reported here for the first time, and the molecular docking analysis provides a scientifically rigorous foundation for understanding potential mechanisms of action, particularly for the sulfated ST **5**. Nonetheless, additional work is required to achieve complete chemical elucidation and reproducibility. Future studies should include the isolation and structural confirmation of pure sulfated STs, especially Compound **5** and derivatives, through further chromatographic purification, followed by in vitro and in vivo validation using standardized antiparasitic assays. These steps will be essential to confirm the bioactivity, assess pharmacological safety, and enable data transparency and reproducibility in alignment with ethical and community research standards.

A limitation of the present study is that the antitrypanosomal activity was evaluated exclusively against the epimastigote stage of *T. cruzi*. Although this developmental form is widely accepted for preliminary screening and provides valuable insights into the trypanocidal potential of crude extracts, it does not represent the clinically relevant intracellular amastigote stage responsible for mammalian infection. Therefore, the observed activity should be interpreted as an initial indicator of antiparasitic potential. Future studies will focus on confirming the efficacy of the most active fractions against intracellular amastigotes in mammalian cell models and, subsequently, in appropriate in vivo systems to further validate their therapeutic relevance.

## 5. Conclusion

This study provides the first chemical and biological characterization of *Isostichopus* sp. aff. *badionotus*, revealing a complex metabolite profile dominated by sulfated STs and other bioactive compounds with potential pharmacological relevance. Among the evaluated fractions, the DCM fraction exhibited the strongest inhibitory activity against *T. cruzi* epimastigotes, which is consistent with its higher content of sulfated sterol derivatives. Molecular docking analyses further supported these findings by indicating favorable binding affinities between the identified sterol compounds and key enzymes involved in the parasite’s redox balance and trypanothione metabolism.

These results were obtained using the epimastigote stage, which is restricted to the intestinal tract of insect vectors and does not represent the clinically relevant intracellular amastigote form responsible for infection in mammalian hosts, including humans, where parasite proliferation occurs predominantly in cardiac and gastrointestinal tissues. Nevertheless, these findings provide a solid preliminary framework for the identification of promising bioactive fractions and support the continued investigation of *Isostichopus* sp. aff. *badionotus* as a source of marine‐derived antitrypanosomal compounds. Future studies will focus on evaluating the activity of the isolated compounds against intracellular amastigotes in mammalian cell models in order to better establish their therapeutic potential.

Our goal was not to assess potential efficacy beyond the in vitro level, as doing so would require a different experimental design and greater material availability. Consequently, an in vivo approach is technically, experimentally, and logistically beyond the scope of this study and is better suited for future studies. In this regard, we believe these findings hold significant value in their own right as a foundational study.

## Author Contributions

Conceptualization: M.E.T‐M., Y.A.R‐N., A.R‐F., and F.E‐A.; investigation: M.E.T‐M., R.A‐I., Y.A.R‐N., E.C‐B., A.R‐F., M.B., J.S.D., L.P‐L., and F.E‐A.; writing—original draft preparation: M.E.T‐M., Y.A.R‐N., E.C‐B., M.B., and F.E‐A.

## Funding

This study was supported by the Ministerio de Ciencia, Tecnología e Innovación (10.13039/100022965, BPIN 2019000100048); Universidad del Magdalena (10.13039/501100016363); FONDECYT (11241068, 3220756); Universidad Militar Nueva Granada (10.13039/501100015812, EXT‐CIAS‐3854).

## Disclosure

All authors have read and agreed to the published version of the manuscript.

## Ethics Statement

This research was approved by the Research Ethics Committee of the Universidad del Magdalena, Colombia, established by Rectoral Resolution No. 427 of 2018. It was also conducted under the Access to Genetic Resources and Derived Products Agreement No. 400, authorized by Resolution No. 1861 of December 26, 2024, between the Ministry of Environment and Sustainable Development and the Universidad del Magdalena. This study does not involve human subjects or animal experimentation.

## Conflicts of Interest

The authors declare no conflicts of interest.

## Data Availability

The datasets generated during and/or analyzed during the current study are available from the corresponding author upon reasonable request.

## References

[1] Lidani K. C. F. , Andrade F. A. , Bavia L. , Damasceno F. S. , Beltrame M. H. , Messias-Reason I. J. , and Sandri T. L. , Chagas Disease: From Discovery to a Worldwide Health Problem, Frontiers in Public Health. (2019) 7, 10.3389/FPUBH.2019.00166, 31312626. PMC6614205 31312626

[2] PAHO/WHO , Chagas Disease, Pan American Health Organization, https://www.paho.org/en/topics/chagas-disease.

[3] Chagas Disease (Also Known as American Trypanosomiasis), https://www.who.int/news-room/fact-sheets/detail/chagas-disease-(american-trypanosomiasis).

[4] Cucunubá Z. M. , Gutiérrez-Romero S. A. , Ramírez J.-D. , Velásquez-Ortiz N. , Ceccarelli S. , Parra-Henao G. , Henao-Martínez A. F. , Rabinovich J. , Basáñez M.-G. , Nouvellet P. , and Abad-Franch F. , The Epidemiology of Chagas Disease in the Americas, Lancet Regional Health–Americas. (2024) 37, 100881, 10.1016/J.LANA.2024.100881, 39474465. 39474465 PMC11519694

[5] Bermudez J. , Davies C. , Simonazzi A. , Pablo Real J. , and Palma S. , Current Drug Therapy and Pharmaceutical Challenges for Chagas Disease, Acta Tropica. (2016) 156, 1–16, 10.1016/J.ACTATROPICA.2015.12.017. 26747009

[6] Mathers C. D. , Ezzati M. , and Lopez A. D. , Measuring the Burden of Neglected Tropical Diseases: The Global Burden of Disease Framework, PLoS Neglected Tropical Diseases. (2007) 1, e114, 10.1371/JOURNAL.PNTD.0000114. 18060077 PMC2100367

[7] Olivera M. J. , Fory J. A. , Porras J. F. , and Buitrago G. , Prevalence of Chagas Disease in Colombia: A Systematic Review and Meta-Analysis, PLoS One. (2019) 14, e0210156, 10.1371/JOURNAL.PONE.0210156. 30615644 PMC6322748

[8] Olivera M. J. , Porras-Villamil J. F. , Villar J. C. , Herrera E. V. , and Buitrago G. , Chagas Disease-Related Mortality in Colombia from 1979 to 2018: Temporal and Spatial Trends, Revista da Sociedade Brasileira de Medicina Tropical. (2021) 54, e0768–2020, 10.1590/0037-8682-0768-2020. 33656153 PMC8008899

[9] Swett M. C. , Rayes D. L. , Campos S. V. , and Kumar R. N. , Chagas Disease: Epidemiology, Diagnosis, and Treatment, Current Cardiology Reports. (2024) 26, no. 10, 1105–1112, 10.1007/S11886-024-02113-7. 39115799

[10] Di Lorenzo Oliveira C. , Nunes M. C. P. , Colosimo E. A. , de Lima E. M. , Cardoso C. S. , Ferreira A. M. , de Oliveira L. C. , Moreira C. H. V. , Bierrenbach A. L. , Ana Haikal D. S. , and Peixoto S. V. , Risk Score for Predicting 2-Year Mortality in Patients With Chagas Cardiomyopathy From Endemic Areas: Sami-Trop Cohort Study, Journal of the American Heart Association. (2020) 9, e014176, 10.1161/JAHA.119.014176. 32157953 PMC7335521

[11] Gulin J. E. N. , Bisio M. M. C. , Rocco D. , Altcheh J. , Solana M. E. , and García-Bournissen F. , Miltefosine and Benznidazole Combination Improve Anti-*Trypanosoma cruzi In Vitro* and *In Vivo* Efficacy, Frontiers in Cellular and Infection Microbiology. (2022) 12, 855119, 10.3389/FCIMB.2022.855119. 35865815 PMC9294734

[12] Fraccaroli L. , Ruiz M. D. , Perdomo V. G. , Clausi A. N. , Balcazar D. E. , Larocca L. , and Carrillo C. , Broadening the Spectrum of Ivermectin: Its Effect on *Trypanosoma cruzi* and Related Trypanosomatids, Frontiers in Cellular and Infection Microbiology. (2022) 12, 885268, 10.3389/FCIMB.2022.885268, 35967842. 35967842 PMC9366347

[13] Almeida-Silva J. , Menezes D. S. , Fernandes J. M. P. , Almeida M. C. , Vasco-dos-Santos D. R. , Saraiva R. M. , Viçosa A. L. , Perez S. A. C. , Andrade S. G. , Suarez-Fontes A. M. , and Vannier-Santos M. A. , The Repositioned Drugs Disulfiram/Diethyldithiocarbamate Combined to Benznidazole: Searching for Chagas Disease Selective Therapy, Preventing Toxicity and Drug Resistance, Frontiers in Cellular and Infection Microbiology. (2022) 12, 926699, 10.3389/FCIMB.2022.926699, 35967878. 35967878 PMC9372510

[14] Benaim G. , Paniz-Mondolfi A. E. , and Sordillo E. M. , The Rationale for Use of Amiodarone and Its Derivatives for the Treatment of Chagas’ Disease and Leishmaniasis, Current Pharmaceutical Design. (2020) 27, 1825–1833, 10.2174/1381612826666200928161403. 32988342

[15] Rivera-Santiago L. , Martínez I. , Arroyo-Olarte R. , Díaz-Garrido P. , Cuevas-Hernandez R. I. , and Espinoza B. , Structural New Data for Mitochondrial Peroxiredoxin From *Trypanosoma cruzi* Show High Similarity With Human Peroxiredoxin 3: Repositioning Thiostrepton as Antichagasic Drug, Frontiers in Cellular and Infection Microbiology. (2022) 12, 907043, 10.3389/FCIMB.2022.907043. 35873171 PMC9301493

[16] Farani P. S. G. , Jones K. M. , and Poveda C. , Treatments and the Perspectives of Developing a Vaccine for Chagas Disease, Vaccines. (2024) 12, 10.3390/VACCINES12080870. PMC11359273 39203996

[17] González-Montero M. C. , Andrés-Rodríguez J. , García-Fernández N. , Pérez-Pertejo Y. , Reguera R. M. , Balaña-Fouce R. , and García-Estrada C. , Targeting Trypanothione Metabolism in Trypanosomatids, Molecules. (2024) 29, 10.3390/MOLECULES29102214. PMC11124245 38792079

[18] Irigoín F. , Cibils L. , Comini M. A. , Wilkinson S. R. , Flohé L. , and Radi R. , Insights Into the Redox Biology of *Trypanosoma cruzi*: Trypanothione Metabolism and Oxidant Detoxification, Free Radical Biology and Medicine. (2008) 45, 733–742, 10.1016/J.FREERADBIOMED.2008.05.028. 18588970

[19] Lo Presti M. S. , Bazán P. C. , Strauss M. , Báez A. L. , Rivarola H. W. , and Paglini-Oliva P. A. , Trypanothione Reductase Inhibitors: Overview of the Action of Thioridazine in Different Stages of Chagas Disease, Acta Tropica. (2015) 145, 79–87, 10.1016/J.ACTATROPICA.2015.02.012. 25733492

[20] Vázquez K. , Paulino M. , Salas C. O. , Zarate-Ramos J. J. , Vera B. , and Rivera G. , Trypanothione Reductase: A Target for the Development of *Anti- Trypanosoma cruzi* Drugs, Mini Reviews in Medicinal Chemistry. (2017) 17, no. 11, 939–946, 10.2174/1389557517666170315145410, 28302040. 28302040

[21] Benítez D. , Franco J. , Sardi F. , Leyva A. , Durán R. , Choi G. , Yang G. , Kim T. , Kim N. , Heo J. , Kim K. , Lee H. , Choi I. , Radu C. , Shum D. , No J. H. , and Comini M. A. , Drug-like Molecules With Anti-Trypanothione Synthetase Activity Identified by High Throughput Screening, Journal of Enzyme Inhibition and Medicinal Chemistry. (2022) 37, no. 1, 912–929, 10.1080/14756366.2022.2045590, 35306933. 35306933 PMC8942522

[22] Tripathi R. K. P. , Dey R. , and Das N. , Identification of Natural Lead Molecules as Potential *Trypanosoma cruzi* Cruzipain Inhibitors and Decoding the Interaction Mechanism for the Treatment of Chagas Disease: A Computational Biology Analysis, Natural Product Research. (2024) 38, no. 20, 3676–3680, 10.1080/14786419.2023.2256018, 37674430. 37674430

[23] Lazarin-Bidóia D. , Garcia F. P. , Ueda-Nakamura T. , Silva S. O. , and Nakamura C. V. , Natural Compounds Based Chemotherapeutic against Chagas Disease and Leishmaniasis: Mitochondrion as a Strategic Target, Memórias do Instituto Oswaldo Cruz. (2022) 117, e220396, 10.1590/0074-02760220396. 35352776 PMC8970591

[24] Izumi E. , Ueda-Nakamura T. , Dias Filho B. P. , Veiga Júnior V. F. , and Nakamura C. V. , Natural Products and Chagas′ Disease: A Review of Plant Compounds Studied for Activity against Trypanosoma Cruzi, Natural Product Reports. (2011) 28, 809–823, 10.1039/C0NP00069H. 21290079

[25] Karthikeyan A. , Joseph A. , and Nair B. G. , Promising Bioactive Compounds From the Marine Environment and Their Potential Effects on Various Diseases, Journal of Genetic Engineering and Biotechnology. (2022) 20, 1–38, 10.1186/S43141-021-00290-4. 35080679 PMC8790952

[26] Mercier A. , Gebruk A. , Kremenetskaia A. , and Hamel J. F. , An Overview of Taxonomic and Morphological Diversity in Sea Cucumbers (Holothuroidea: Echinodermata), World of Sea Cucumbers: Challenges, Advances, and Innovations. (2024) 3–15, 10.1016/B978-0-323-95377-1.00001-1.

[27] Mohammadizadeh F. , Ehsanpor M. , Afkhami M. , Mokhlesi A. , Khazaali A. , and Montazeri S. , Evaluation of Antibacterial, Antifungal and Cytotoxic Effects of *Holothuria scabra* From the North Coast of the Persian Gulf, Journal de Mycologie Médicale. (2013) 23, 225–229, 10.1016/J.MYCMED.2013.08.002. 24054089

[28] Janakiram N. B. , Mohammed A. , and Rao C. V. , Sea Cucumbers Metabolites as Potent Anti-Cancer Agents, Marine Drugs. (2015) 13, 2909–2923, 10.3390/MD13052909. 25984989 PMC4446612

[29] Baharara J. , Amini E. , Kerachian M. A. , and Soltani M. , The Osteogenic Differentiation Stimulating Activity of Sea Cucumber Methanolic Crude Extraction on Rat Bone Marrow Mesenchymal Stem Cells, Iranian Journal of Basic Medical Sciences. (2014) 17. PMC4240799 25422758

[30] Vergara W. , Agudelo V. , Castro L. R. , Rodríguez A. , and Eeckhaut I. , Morphological and Molecular Characterization of *Isostichopus* sp. in the Colombian Caribbean Sea, Journal of Basic and Applied Genetics. (2018) 29, 33–48, 10.35407/BAG.2018.29.02.04.

[31] Taborda-Martínez M. E. , Rodríguez-Forero A. , Bacho M. , Espitia-Almeida F. , Coy-Barrera E. , and Rodríguez-Núñez Y. A. , Metabolite Profiling-Based Characterization and Antibacterial Activity of Sea Cucumber *Isostichopus*. sp. *Aff. Badionotus* from Colombian Caribbean Sea, 2025, Preprints (Basel). 10.1177/00368504251407127 PMC12743769 41432621

[32] Vergara Hernández W. and Rodríguez A. , Histology of the Digestive Tract of Three species of Sea Cucumber *Isostichopus badionotus*, *Isostichopus* sp. and *Stichopus hermanni* (Aspidochirotida: Stichopodidae), Revista de Biología Tropical. (2015) 63, no. 4, 1021–1033.

[33] Cázares-Jaramillo G. E. , Molina-Garza Z. J. , Luna-Cruz I. E. , Solís-Soto L. Y. , Rosales-Encina J. L. , and Galaviz-Silva L. , *In Vitro* Anti-*Trypanosoma cruzi* Activity of Methanolic Extract of *Bidens pilosa* and Identification of Active Compounds by Gas Chromatography-Mass Spectrometry Analysis, Parasites, Hosts and Diseases. (2023) 61, 405–417, 10.3347/PHD.23069. 38043536 PMC10693971

[34] Pérez-Treviño K. C. , Galaviz L. , Iracheta-Villarreal J. M. , Lucero-Velasco E. A. , and Molina-Garza Z. J. , Activity Against *Trypanosoma cruzi* (Kinetoplastida: Trypanosomatidae) of Methanolic Extracts of Medicinal Use Plants in Mexico, Revista de Biología Tropical. (2017) 65, 1459–1469, 10.15517/rbt.v65i4.27153.

[35] Castañeda J. S. , Suta-Velásquez M. , Mateus J. , Pardo-Rodriguez D. , Puerta C. J. , Cuéllar A. , Robles J. , and Cuervo C. , Preliminary Chemical Characterization of Ethanolic Extracts From Colombian Plants With Promising Anti-*Trypanosoma cruzi* Activity, Experimental Parasitology. (2021) 223, 108079, 10.1016/j.exppara.2021.108079. 33524381

[36] Espitia-Almeida F. , Diaz-Uribe C. , Vallejo W. , Gómez-Camargo D. , Romero Bohórquez A. R. , and Linares-Flores C. , Photophysical Study and *In Vitro* Approach Against *Leishmania panamensis* of Dicloro-5,10,15,20-Tetrakis(4-Bromophenyl)Porphyrinato Sn(IV), F1000Research. (2021) 10, 10.12688/f1000research.52433.3. PMC8581593 34804494

[37] Espitia-Almeida F. , Díaz-Uribe C. , Vallejo W. , Gómez-Camargo D. , and Romero Bohórquez A. R. , *In Vitro* Anti-Leishmanial Effect of Metallic Meso-Substituted Porphyrin Derivatives Against *Leishmania braziliensis* and *Leishmania panamensis* Promastigotes Properties, Molecules. (2020) 25, 10.3390/MOLECULES25081887. PMC7221524 32325815

[38] Schymanski E. L. , Jeon J. , Gulde R. , Fenner K. , Ruff M. , Singer H. P. , and Hollender J. , Identifying Small Molecules via High Resolution Mass Spectrometry: Communicating Confidence, Environtal Science & Technology. (2014) 48, 2097–2098, 10.1021/es5002105. 24476540

[39] Morris G. M. , Ruth H. , Lindstrom W. , Sanner M. F. , Belew R. K. , Goodsell D. S. , and Olson A. J. , AutoDock4 and AutoDockTools4: Automated Docking With Selective Receptor Flexibility, Journal of Computational Chemistry. (2009) 30, 2785–2791, 10.1002/JCC.21256. 19399780 PMC2760638

[40] Sowerby K. , Freitag-Pohl S. , Murillo A. M. , Silber A. M. , and Pohl E. , Cysteine Synthase: Multiple Structures of a Key Enzyme in Cysteine Synthesis and a Potential Drug Target for Chagas Disease and Leishmaniasis, Acta Crystallographica D Structural Biology. (2023) 79, 518–530, 10.1107/S2059798323003613/DI5064SUP1.PDF. 37204818 PMC10233618

[41] Bond C. S. , Zhang Y. , Berriman M. , Cunningham M. L. , Fairlamb A. H. , and Hunter W. N. , Crystal Structure of *Trypanosoma cruzi* Trypanothione Reductase in Complex With Trypanothione, and the Structure-Based Discovery of New Natural Product Inhibitors, Structure. (1999) 7, 81–89, 10.1016/S0969-2126(99)80011-2. 10368274

[42] Yoshino R. , Yasuo N. , Hagiwara Y. , Ishida T. , Inaoka D. K. , Amano Y. , Tateishi Y. , Ohno K. , Namatame I. , Niimi T. et al., Discovery of a Hidden *Trypanosoma cruzi* Spermidine Synthase Binding Site and Inhibitors through In Silico, *In Vitro*, and X-Ray Crystallography, ACS Omega. (2023) 8, 25850–25860, 10.1021/ACSOMEGA.3C01314. 37521650 PMC10373461

[43] Janson G. and Paiardini A. , PyMod 3: A Complete Suite for Structural Bioinformatics in PyMOL, Bioinformatics. (2021) 37, 1471–1472, 10.1093/BIOINFORMATICS/BTAA849. 33010156

[44] Rosignoli S. , Lustrino E. , Di Silverio I. , and Paiardini A. , Making Use of Averaging Methods in MODELLER for Protein Structure Prediction, International Journal of Molecular Sciences. (2024) 25, 10.3390/IJMS25031731. PMC10855553 38339009

[45] Laskowski R. A. , MacArthur M. W. , Moss D. S. , and Thornton J. M. , PROCHECK: A Program to Check the Stereochemical Quality of Protein Structures, Applied Crystallography. (1993) 26, 283–291, 10.1107/S0021889892009944.

[46] Salentin S. , Schreiber S. , Haupt V. J. , Adasme M. F. , and Schroeder M. , PLIP: Fully Automated Protein-Ligand Interaction Profiler, Nucleic Acids Research. (2015) 43, W443–W447, 10.1093/NAR/GKV315. 25873628 PMC4489249

[47] Saudagar P. and Dubey V. K. , Molecular Mechanisms of *In Vitro* Betulin-Induced Apoptosis of *Leishmania donovani* , American Journal of Tropical Medicine and Hygiene. (2014) 90, 10.4269/AJTMH.13-0320. PMC3919248 24420777

[48] Saccoliti F. , Di Santo R. , and Costi R. , Recent Advancement in the Search of Innovative Antiprotozoal Agents Targeting Trypanothione Metabolism, ChemMedChem. (2020) 15, 2420–2435, 10.1002/CMDC.202000325. 32805075

[49] Makarieva T. N. , Stonik V. A. , Kapustina I. I. , Boguslavsky V. M. , Dmitrenoik A. S. , Kalinin V. I. , Cordeiro M. L. , and Djerassi C. , Biosynthetic Studies of Marine Lipids. 42. Biosynthesis of Steroid and Triterpenoid Metabolites in the Sea Cucumber *Eupentacta fraudatrix* , Steroids. (1993) 58, 508–517, 10.1016/0039-128X(93)90026-J. 8273112

[50] Goad L. J. , Garneau F.-X. , Simard J. L. , Apsimon J. W. , and Girard M. , Composition of the Free, Esterified and Sulphated Sterols of the Sea Cucumber *Psolus fabricii* , Comparative Biochemistry and Physiology Part B: Comparative Biochemistry. (1986) 84, 189–196, 10.1016/0305-0491(86)90204-X.

[51] Srihera N. , Li Y. , Zhang T.-T. , Wang Y.-M. , Yanagita T. , Waiprib Y. , and Xue C.-H. , Preparation and Characterization of Astaxanthin-Loaded Liposomes Stabilized by Sea Cucumber Sulfated Sterols Instead of Cholesterol, Journal of Oleo Science. (2022) 71, 401–410, 10.5650/jos.ess21233. 35153245

[52] Ding L. , Xu Z.-J. , Shi H.-H. , Xue C.-H. , Huang Q.-R. , Yanagita T. , Wang Y.-M. , and Zhang T.-T. , Sterol Sulfate Alleviates Atherosclerosis via Mediating Hepatic Cholesterol Metabolism in ApoE−/− Mice, Food & Function. (2021) 12, 4887–4896, 10.1039/D0FO03266B. 33977967

[53] Zhang H.-J. , Chen C. , Ding L. , Shi H.-H. , Wang C.-C. , Xue C.-H. , Zhang T.-T. , and Wang Y.-M. , Sea Cucumbers-Derived Sterol Sulfate Alleviates Insulin Resistance and Inflammation in High-Fat-High-Fructose Diet-Induced Obese Mice, Pharmacological Research. (2020) 160, 105191, 10.1016/j.phrs.2020.105191. 32911073

[54] Zhou X. , Zhou D.-Y. , Yin F.-W. , Song L. , Liu Y.-X. , Xie H.-K. , Gang K.-Q. , Zhu B.-W. , and Shahidi F. , Glycerophospholipids in Sea Cucumber (*Stichopus japonicus*) and Its Processing By-Products Serve as Bioactives and Functional Food Ingredients, Journal of Food Bioactives. (2018) 1, 134–142, 10.31665/JFB.2018.1132.

[55] Pardo-Rodriguez D. , Cifuentes-López A. , Bravo-Espejo J. , Romero I. , Robles J. , Cuervo C. , Mejía S. M. , and Tellez J. , Lupeol Acetate and *α*-Amyrin Terpenes Activity Against *Trypanosoma cruzi*: Insights Into Toxicity and Potential Mechanisms of Action, Tropical Medicine and Infectious Disease. (2023) 8, 10.3390/tropicalmed8050263. PMC10220761 37235311

[56] Porta E. O. J. , Ballari M. S. , Carlucci R. , Wilkinson S. , Ma G. , Tekwani B. L. , and Labadie G. R. , Systematic Study of 1,2,3-Triazolyl Sterols for the Development of New Drugs against Parasitic Neglected Tropical Diseases, European Journal of Medicinal Chemistry. (2023) 254, 115378, 10.1016/j.ejmech.2023.115378. 37084599

[57] Pereira C. G. , Moraes C. B. , Franco C. H. , Feltrin C. , Grougnet R. , Barbosa E. G. , Panciera M. , Correia C. R. D. , Rodrigues M. J. , and Custódio L. , *In Vitro* Anti-*Trypanosoma cruzi* Activity of Halophytes From Southern Portugal Reloaded: A Special Focus on Sea Fennel (*Crithmum maritimum* L.), Plants. (2021) 10. 10.3390/plants10112235 PMC8625203 34834598

[58] Tibayrenc M. and Ayala F. J. , The Clonal Theory of Parasitic Protozoa: 12 Years On, Trends in Parasitology. (2002) 18, 405–410, 10.1016/S1471-4922(02)02357-7. 12377258

[59] Morel C. , Chiari E. , Camargo E. P. , Mattei D. M. , Romanha A. J. , and Simpson L. , Strains and Clones of *Trypanosoma cruzi* Can Be Characterized by Pattern of Restriction Endonuclease Products of Kinetoplast DNA Minicircles, Proceedings of the National Academy of Sciences. (1980) 77, 6810–6814, 10.1073/pnas.77.11.6810. PMC350379 6256762

[60] Llovera A. , Abras A. , Fernández-Arévalo A. , Ballart C. , Heras S. , Muñoz C. , and Gállego M. , Genetic Diversity of *Trypanosoma cruzi* in the United States of America: The Least Endemic Country for Chagas Disease, Life. (2024) 14. 10.3390/life14070901 PMC11278504 39063654

[61] Pounina T. A. , Gloriozova T. A. , Savidov N. , and Dembitsky V. M. , Sulfated and Sulfur-Containing Steroids and Their Pharmacological Profile, Marine Drugs. (2021) 19, 10.3390/md19050240. PMC8145587 33923288

[62] Begum R. , Howlader S. , Mamun-Or-Rashid A. N. M. , Rafiquzzaman S. M. , Ashraf G. M. , Albadrani G. M. , Sayed A. A. , Peluso I. , Abdel-Daim M. M. , and Uddin M. S. , Antioxidant and Signal-Modulating Effects of Brown Seaweed-Derived Compounds Against Oxidative Stress-Associated Pathology, Oxidative Medicine and Cellular Longevity. (2021) 2021, 9974890, 10.1155/2021/9974890. 34336128 PMC8289617

[63] Gómez-Escobedo R. , Méndez-Álvarez D. , Vázquez C. , Saavedra E. , Vázquez K. , Alcántara-Farfán V. , Cordero-Martínez J. , Gonzalez-Gonzalez A. , Rivera G. , and Nogueda-Torres B. , Molecular Docking-Based Virtual Screening of FDA-Approved Drugs Using Trypanothione Reductase Identified New Trypanocidal Agents, Molecules. (2024) 29, 10.3390/MOLECULES29163796/S1. PMC11357579 39202874

[64] Bernal F. A. and Coy-Barrera E. , In-Silico Analyses of Sesquiterpene-Related Compounds on Selected *Leishmania* Enzyme-Based Targets, Molecules. (2014) 19, 5550–5569, 10.3390/MOLECULES19055550. 24786692 PMC6271876

